# *CTLA4* promoter methylation predicts response and progression-free survival in stage IV melanoma treated with anti-CTLA-4 immunotherapy (ipilimumab)

**DOI:** 10.1007/s00262-020-02777-4

**Published:** 2020-11-16

**Authors:** Simon Fietz, Romina Zarbl, Dennis Niebel, Christian Posch, Peter Brossart, Gerrit H. Gielen, Sebastian Strieth, Torsten Pietsch, Glen Kristiansen, Friedrich Bootz, Jennifer Landsberg, Dimo Dietrich

**Affiliations:** 1 Department of Dermatology and Allergy, University Hospital Bonn, Venusberg-Campus, Bonn, 53127 Germany; 2 Department of Otolaryngology, Head and Neck Surgery, University Hospital Bonn, Venusberg-Campus, 53127 Bonn, Germany; 3 Department of Dermatology and Allergy, Technical University of Munich, Munich, Germany; 4 Faculty of Medicine, Sigmund Freud University, Vienna, Austria; 5 Department of Oncology, Hematology and Rheumatology, University Hospital Bonn, Bonn, Germany; 6 Institute of Neuropathology, University Hospital Bonn, Bonn, Germany; 7 Institute of Pathology, University Hospital Bonn, Bonn, Germany

**Keywords:** CTLA-4, *CTLA4*, DNA methylation, Immunotherapy, Predictive biomarker, Melanoma

## Abstract

Anti-CTLA-4-antibodies can induce long-lasting tumor remissions. However, only a few patients respond, necessitating the development of predictive companion biomarkers. Increasing evidence suggests a major role of epigenetics, including DNA methylation, in immunology and resistance to immune checkpoint blockade. Here, we tested *CTLA4* promoter methylation and CTLA-4 protein expression as predictive biomarkers for response to anti-CTLA-4 immunotherapy. We identified retrospectively *N* = 30 stage IV melanoma patients treated with single-agent anti-CTLA-4 immunotherapy (ipilimumab). We used quantitative methylation-specific PCR and immunohistochemistry to quantify *CTLA4* methylation and protein expression in pre-treatment samples. *CTLA4* methylation was significantly higher in progressive as compared to responding tumors and significantly associated with progression-free survival. A subset of infiltrating lymphocytes and tumor cells highly expressed CTLA-4. However, CTLA-4 protein expression did not predict response to treatment. We conclude that *CTLA4* methylation is a predictive biomarker for response to anti-CTLA-4 immunotherapy.

## Introduction

Therapeutic monoclonal antibodies (mAbs) targeting the immune checkpoints cytotoxic T lymphocyte-associated protein 4 (CTLA-4) and programmed cell death 1 (PD-1) have revolutionized the treatment of various cancers. In metastatic melanoma immune checkpoint blockade (ICB) considerably prolongates survival and even leads to durable remission in some cases [1, 2]. However, only a subgroup of patients responds to treatment due to primary resistance to ICB. This is particularly true for ipilimumab, the first in class CTLA-4-directed immune checkpoint inhibitor, which has shown efficacy in 19% of patients [2]. Compared to anti-PD-1 inhibitors, e.g., nivolumab and pembrolizumab, the efficacy of ipilimumab is lower and accompanied with 30% more high-grade side effects than nivolumab [2]. Since a small group of patients shows dramatic responses to ipilimumab monotherapy, CTLA-4 remains an important immunotherapy target. Hence, robust biomarkers indicating patients, who benefit from anti-CTLA-4 first-line monotherapy, are needed in the field of personalized medicine. Such biomarkers are of considerable interest since next-generation CTLA-4-directed compounds (mono- or bispecific antibodies, probodies) are under investigation in preclinical up to phase III trials. According to preclinical trials, ipilimumab probodies are believed to show comparable efficacy with less adverse reactions [3]. Therefore, it constitutes a promising companion drug in the treatment of advanced melanoma. Ongoing biomarker research mainly focuses on response data of patients treated with anti-PD-1 mAbs [4]. Of particular interest are parameters of the tumor microenvironment, such as tumor-infiltrating immune cells (TILs), immune checkpoint (mainly PD-L1) expression, and immune gene signatures [5]. However, genetic alterations, for instance, microsatellite instability (MSI), mutational and neoantigen burden are also actively investigated [6, 7]. Epigenetic alterations, however, have widely been neglected as potential predictive biomarkers so far but have great potential due to their significance in tumorigenesis and immunology, including immune cell differentiation and T cell exhaustion [8–12]. Recent reports suggest DNA methylation of genes encoding for the immune checkpoints 4-1BB, LAG-3, PD-L2, and CTLA-4 as predictive biomarkers for response to ICB in melanoma [13–16]. Our present study aimed at the analysis of the value of *CTLA4* methylation as a biomarker to predict response and progression under CTLA-4-directed ICB monotherapy. In addition, we compared *CTLA4* methylation with CTLA-4 protein expression as a potential predictive biomarker.

## Materials and methods

Our study comprised histologically confirmed pretreatment melanomas from a cohort of *N* = 30 stage IV melanoma patients treated with anti-CTLA-4 antibody ipilimumab as first-line immunotherapy at the University Hospital Bonn and Technical University of Munich between 2010 and 2015. The patients received 2–6 doses (3 mg/kg body weight) ipilimumab. Median number of applications was four. Our study was approved by the Institutional Review Board (IRB) of the University Hospital Bonn (vote no. 187/16). Patient and sample characteristics are described in detail in Table 1. We analyzed PFS and response as clinical endpoint. PFS was defined as time between the first application of anti-CTLA-4 ICB and the date of documented disease progress or last contact, respectively. Response was determined in accordance with RECIST version 1.1.

**Table 1:** Patient characteristics at baseline and their associations with PFS, response, and *CTLA4* promoter methylation

Characteristic	Total cohort (*N* = 30)	PFS		*CTLA4* methylation		Response	
		Hazard ratio [95% CI]	*P* value	Mean (%), [SD]	*P* value	Responder (*N* = 6)	Non-responder (*N* = 24)
**Median age (range)—years**	64 (28–86)					65 (49–76)	64 (28–86)
**Sex—no. (%)**					*P* = 0.54		
Male	17 (57)	Ref group		22.8 [30.5]		5 (83)	12 (50)
Female	13 (43)	2.58 [1.13–5.87]	*P* = 0.024	17.5 [19.7]		1 (17)	12 (50)
**Disease origin—no. (%)**			*P* = 0.13		*P* = 0.13		
Cutaneous	20 (67)	Ref group		17.2 [18.1]		4 (67)	16 (67)
Acral	2 (7)	1.67 [0.37–7.54]	*P* = 0.50	23.2 [3.9]		0 (0)	2 (8)
Uveal	1 (3)	1.01 [0.13–7.87]	*P* = 0.99	24.7		0 (0)	1 (4)
Meningeal	1 (3)	0.69 [0.09–5.11]	*P* = 0.70	130.4		0 (0)	1 (4)
Melanoma of unknown primary (MUP)	2 (7)	11.2 [1.81–69.2]	*P* = 0.009	7.8 [6.7]		0 (0)	2 (8)
Not available	4 (13)	NA	NA	NA		2 (33)	2 (8)
**Stage at baseline—no. (%)**			*P* = 0.59		*P* = 0.79		
M1a	3 (10)	2.49 [0.60–10.3]	*P* = 0.21	11.9 [8.7]		0 (0)	3 (13)
M1b	3 (10)	1.57 [0.41–6.11]	*P* = 0.51	16.2 [8.7]		1 (17)	2 (8)
M1c	13 (43)	1.74 [0.65–4.69]	*P* = 0.27	21.6 [21.7]		1 (17)	12 (50)
M1d	10 (33)	Ref group		21.6 [38.4]		4 (67)	6 (25)
Unknown	1 (3)	NA	NA	NA		0 (0)	1 (4)
**Brain metastases at baseline— no. (%)**					*P* = 0.46		
No	19 (63)	Ref group		19.2 [18.6]		2 (33)	17 (71)
Yes	10 (33)	0.56 [0.23–1.40]	*P* = 0.22	21.6 [38.4]		4 (67)	6 (25)
Unknown	1 (3)	NA	NA	NA		0 (0)	1 (4)
**BRAF mutation—no. (%)**					*P* = 0.22		
Yes	5 (17)	5.15 [1.43–18.5]	*P* = 0.012	17.8 [9.1]		0 (0)	5 (21)
No	23 (77)	Ref group		21.4 [29.5]		5 (83)	18 (75)
Not available	2 (7)	NA	NA	NA		1 (17)	1 (4)
**NRAS** **mutation—no. (%)**					*P* = 0.24		
Yes	14 (47)	0.98 [0.97–1.00]	*P* = 0.97	13.7 [12.0]		3 (50)	11 (46)
No	6 (20)	Ref group		27.9 [27.0]		1 (17)	5 (21)
Not available	10 (33)	NA	NA	NA		2 (33)	8 (33)
**LDH at baseline—no. (%)**					*P* = 0.72		
Normal	16 (53)	Ref group		20.6 [31.4]		5 (83)	11 (46)
Elevated	11 (37)	3.08 [1.14–8.30]	*P* = 0.026	18.3 [20.2]		0 (0)	11 (46)
Not available	3 (10)	NA	NA	NA		1 (17)	2 (8)
**Previous therapies—no. (%)**^§^			*P* = 0.59		*P* = 0.46		
Chemotherapy	7 (23)	0.86 [0.35–2.24]	*P* = 0.80	21.5 [27.6]		1 (17)	6 (25)
Targeted therapy	2 (7)	2.07 [0.46–9.31]	*P* = 0.34	9.7 [1.6]		0 (0)	2 (8)
None	21 (70)	Ref group		20.6 [27.8]		5 (83)	16 (67)
**Sample origin—no. (%)**			*P* = 0.65		*P* = 0.48		
Skin	10 (33)	Ref group		23.9 [22.9]		1 (17)	9 (38)
Lymph node	10 (33)	0.69 [0.26–1.81]	*P* = 0.45	11.1 [8.0]		2 (33)	8 (33)
Lung	4 (13)	0.63 [0.19–2.09]	*P* = 0.45	9.5 [3.6]		1 (17)	3 (13)
Liver	2 (7)	0.82 [0.17–3.89]	*P* = 0.80	26.3 [22.3]		0 (0)	2 (8)
Brain	3 (10)	0.24 [0.05–1.14]	*P* = 0.073	49.9 [69.7]		2 (33)	1 (4)
Uvea	1 (3)	0.74 [0.09–5.98]	*P* = 0.78	24.7		0 (0)	1 (4)
**Response—no. (%)**			ND		*P* = 0.052		
Objective response^¥^	6 (20)	ND	ND	7.6 [2.3]		6 (100)	0 (0)
Progressive disease	21 (70)	Ref group		23.7 [29.9]		0 (0)	21 (86)
Stable disease	3 (10)	0.30 [0.07–1.33]	*P* = 0.11	24.6 [16.1]		0 (0)	3 (13)
**TILs—no. (%)**					*P* = 0.086		
Non-brisk/brisk	20 (67)	0.79 [0.36–1.76]	*P* = 0.57	11.7 [6.2]		4 (67)	16 (67)
Absent	10 (33)	Ref group		38.1 [39.9]		2 (33)	8 (33)
**CTLA-4**^+^ **tumor cells—no. (%)**			*P* = 0.24		*P* = 0.29		
H score ≥ 200	6 (20)	1.73 [0.63–4.73]	*P* = 0.28	17.5 [11.2]		1 (17)	5 (21)
H score100-199	10 (33	2.17 [0.85–5.50]	*P* = 0.10	23.0 [22.4]		1 (17)	9 (38)
H score ≤ 99	14 (47)	Ref group		20.0 [33.3]		4 (67)	10 (42)

*NA *not analyzed

^§^Previous therapies included chemotherapy and targeted therapy

^¥^This category included patients with a complete response (*N* = 3) and those with a partial response (*N* = 3)

*ND *not determined (hazard ratio and *P* values could not be calculated because of complete separation of the predictor variable CR/PR)

For methylation analysis, we used FFPE tumor tissues mounted on glass slides. After tumor macrodissection, we applied the innuCONVERT Bisulfite All-In-One Kit (Analytik Jena, Jena, Germany) for tissue lysis, bisulfite conversion, and DNA purification following the manufacturer’s protocol. We performed qMSP to evaluate *CTLA4* promoter methylation as previously described [16]. In brief, we determined relative *CTLA4* methylation levels referred to total DNA in duplex qMSP reactions. A CpG-free target region within the housekeeping gene *ACTB* was used as reference.

Prior to immunohistochemistry, 4-µm FFPE tumor tissues sections were deparaffinizated with xylene, rehydrated through a descending ethanol series, and finally washed with 550 mM Tris-buffered saline (TBS). Heavily pigmented melanoma sections were bleached with 30% H_2_O_2_ and 0.5% potassium hydroxide for 30 min at 37 °C. For antigen retrieval, the sections were incubated with Target Retrieval Solution (pH6, Dako/ Agilent Technologies, Inc., Santa Clara, CA, USA) at 100 °C for 10 min and were washed with TBS subsequently. Primary CTLA-4 antibody (dilution 1:50, mouse monoclonal antibody, clone: BSB-88, Bio SB, Santa Barbara, CA, USA) was added, incubated at 4 °C overnight, and subsequently washed with 550 mM TBS. REAL Detection System Alkaline Phosphatase/RED (Dako/Agilent Technologies) was utilized to visualize bounded primary antibody according to the manufacturer’s protocol. Finally, we used Mayer’s Hemalum solution (Merck Millipore, Billerica, MA, USA) to contrast the staining. CTLA-4 expression by tumor cells was quantified using the H scoring system [17]. Tumor-infiltrating lymphocytes were assessed using the scoring system by Clark: absent = no TILs, non-brisk = focal TILs, brisk = diffuse TILs [18]. CTLA-4-expressing immune cells were analyzed qualitatively.

Statistical tests were performed utilizing SPSS, version 23.0 (SPSS Inc., Chicago, IL). Kaplan–Meier and Cox proportional hazards regression analyses were conducted (*P* values refer to log-rank and Wald tests, respectively). Survival analyses were performed using dichotomized methylation levels and categorized variates, respectively. Mann–Whitney *U* test (2 groups) and Kruskal–Wallis (> 2 groups) test were applied for arithmetical mean comparison. Correlations were computed using Spearman’s rank correlation (Spearman’s *ρ*). Two-sided *P* values < 0.05 were considered statistically significant.

## Results

### Analyses of *CTLA4* methylation in *N* = 30 FFPE melanoma samples from patients prior ipilimumab therapy

To test the utility of *CTLA4* methylation as a predictive biomarker for response to anti-CTLA-4 immune checkpoint blockade in stage IV melanoma patients, we identified retrospectively 30 patients diagnosed with advanced melanoma and treated with anti-CTLA-4 antibody ipilimumab monotherapy. Median time from biopsy to initiation of anti-CTLA-4 blockade was 7 months. Best objective response to anti-CLTA-4 therapy using the Response Evaluation Criteria in Solid Tumors (RECIST) version 1.1 included 3/30 (10%) with complete response (CR), 3/30 (10%) with partial response (PR), 3/30 (10%) with stable disease (SD), and 21/30 (70%) with progressive disease (PD). All patients that achieved complete response had brain metastases and received ipilimumab without radiation therapy. Two of these patients remained tumor-free (5.3 years and 5.9 years follow-up time). Overall response rate was 20% (CR + PR). 9/30 (30%) patients were previously treated with targeted therapy or chemotherapy, whereas 21/30 (70%) were therapy naïve. Median follow-up for survival was 3 months. Clinical characteristics are described in detail in Table 1. We performed a quantitative methylation-specific PCR assay (qMSP) targeting the CpG-site of interest using DNA from formalin-fixed and paraffin-embedded (FFPE) melanoma samples prior ipilimumab treatment.

### Association of *CTLA4* methylation with response to ipilimumab and PFS

**Fig. 1:**
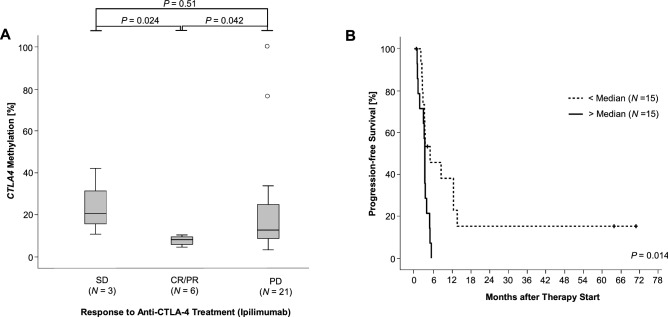
Association of *CTLA4* promoter methylation with response and PFS in melanoma patients treated with ipilimumab. **a** *CTLA4* methylation levels in melanomas from patients with stable disease (SD), complete or partial response (CR/PR), and progressive disease (PD). **b** Kaplan–Meier analyses of PFS in melanoma patients receiving anti-CTLA-4 monotherapy (ipilimumab)

First, we tested the association of *CTLA4* promoter methylation with response according to RECIST version 1.1. Despite the small sample size, we could find significantly lower methylation levels in melanoma samples from responders [complete and partial responders; mean methylation level 7.6 ± 2.3, 6/30 (20%)] compared to patients with progressive disease (mean methylation level 23.7 ± 29.9, 21/30 (70%), *P* = 0.042) and to patients with stable disease (mean methylation level 24.4 ± 16.1, 3/30 (10%), *P* = 0.024, Fig. 1a). We did not find significant methylation differences between samples from patients with stable disease compared to patients with progressive disease.

We further tested the association of *CTLA4* promoter methylation with progression-free survival (PFS). We classified patient samples according to the median methylation (10.9%) of the whole cohort as *CTLA4* methylation above median and below median, respectively. We used median methylation as cutoff for dichotomization of methylation levels to avoid an overfitted model due to the introduction of an optimized cutoff. Patients with tumor methylation levels below median showed a significantly better PFS as compared to patients with methylation levels above cutoff (*P* = 0.014, Fig. 1b). Patients with low methylated tumors had a PFS of 33% after 1 year (40% after 6 months and 13% after 5 years) while all patients with highly methylated melanomas progressed within 6 months after immunotherapy start.

### Association of clinical, molecular, and histomorphological features with PFS and *CTLA4* methylation

Significantly worse PFS was associated with female sex, *BRAF*-mutated tumors, and patients with increased LDH levels at baseline (Table 1). We found no significant association between TILs and PFS or response to ipilimumab. However, we found a trend towards lower methylation levels in melanomas with brisk/non-brisk TILs (mean methylation level 11.7 ± 6.2, 20/30 (67%)) compared to melanomas with no TILs (mean methylation level 38.1 ± 39.9, 10/30 (33%), *P* = 0.086, Table 1).

### Association of *CTLA4* methylation and CTLA-4 protein expression

**Fig. 2:**
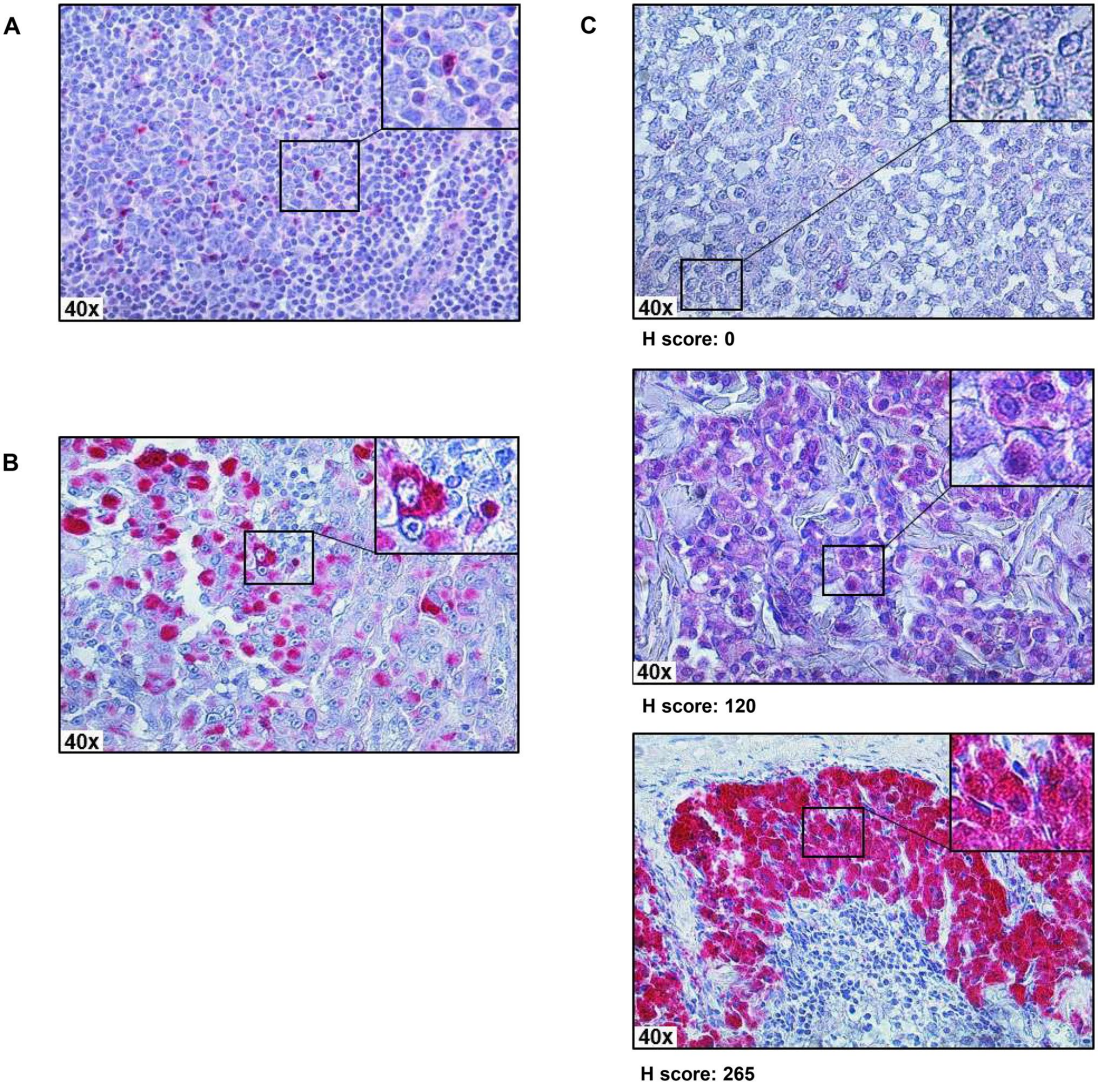
CTLA-4 protein expression in tonsillar and melanoma tissue. Immunohistochemical staining of CTLA-4 in a tonsil (**a**) and exemplarily in four melanomas (**b**, **c**). CTLA-4-expressing lymphocytes are present in tonsillar tissue (**a**). Melanoma with CTLA-4-expressing tumor and immune cells (**b**). Melanomas with mainly CTLA-4-negative, weakly CTLA-4-positive, and strongly CTLA-4-positive tumor cells, respectively, and corresponding H scores (**c**)

We have previously shown that *CTLA4* promoter methylation inversely correlates with its corresponding mRNA expression (Spearman’s *ρ* =  – 0.42, *P* < 0.001) in a large melanoma cohort from The Cancer Genome Atlas [16]. Following up on this finding and to dissect melanoma or immune cells as source of CTLA-4 expression, we performed IHC for CTLA-4 protein expression in our melanoma cohort prior ipilimumab treatment. As expected, positive CTLA-4 IHC staining was found for a subset of lymphocytes in tonsillar tissue used as a positive control (Fig. 2a). In melanomas, we found CTLA-4 protein expressed predominantly on tumor cells and only on a small subset of infiltrating immune cells (Fig. 2b). CTLA-4 expression by melanoma cells was heterogeneous, e.g., tumors with predominantly CTLA-4-negative tumor cells, tumors with mainly weakly CTLA-4-expressing tumor cells, and melanomas with strongly CTLA-4-expressing tumor cells (Fig. 2c). Quantitative CTLA-4 protein expression as determined using the H scoring system did not reveal a significant correlation between methylation and protein expression (Spearman’s *ρ* = 0.21, *P* = 0.27). Only a subset of tumors contained CTLA-4^+^ immune cells (40%, 12/30). We could not find a correlation between *CTLA4* methylation and the presence of CTLA-4^+^ immune cells (*P* = 0.91). In concordance with the lack of a significant correlation between methylation and protein expression, we did not find significant differences in Kaplan–Meier analysis of PFS in our cohort stratified by CTLA-4 protein-expressing tumor cells (*P* = 0.17) or TILs (*P* = 0.26), respectively.

## Discussion

We have previously reported the utility of *CTLA4* promoter methylation as a predictive biomarker for response to immunotherapy and survival in a heterogeneous cohort comprised of *N* = 50 melanoma patients who received anti-PD-1 and anti-CTLA-4 single-agent or combination immunotherapy [16]. In the present study, we have successfully validated these findings in a homogeneous independent cohort of *N* = 30 melanoma patients treated with CTLA-4-targeted ICB (ipilimumab). We observed an objective response rate of 20% (6/30 patients). This is in accordance with the objective response rate of 19% in the CheckMate067 trial [2]. We found that significantly worse PFS was associated with *BRAF*-mutated tumors, patients with increased LDH baseline levels, and female patients. These findings are also in concordance with other studies analyzing high-stage melanoma patients treated with anti-CTLA-4 monotherapy [19–21].

CTLA-4 protein was mainly expressed by melanoma cells and by a subset of tumor-infiltrating immune cells. The exact mode of action of anti-CTLA-4 ICB and the role of tumor cell-intrinsic CTLA-4 expression with regard to responsiveness to ICB is still only poorly understood. A study by Daud et al. [22] revealed that an increase in the fraction of a certain type of CD8^+^ T cells with high expression of PD-1 and CTLA-4 (PD-1^high^/CTLA-4^high^) in metastatic melanoma correlates with response to anti-PD-1 treatment. Further analysis indicated a partially exhausted phenotype (PD-1^high^/CTLA-4^high^) suggesting that ICB might invigorate particularly PD-1^high^/CTLA-4^high^ CD8^+^ T cells. On tumor cells, expression of PD-L1 could be observed in multiple malignancies and seems to be associated with abundance of TILs [5] and interferon γ response signatures [23]. CTLA-4 protein expression by melanoma cells could be shown by Mo et al. [24]. The role of CTLA-4 in tumor cells, however, is barely understood. In the same study, a survival benefit under immunotherapy was suggested in patients whose tumors showed an interferon γ signature that was associated with a high expression of CTLA-4 mRNA. The predictive value of CTLA-4 mRNA and protein expression in melanoma remains ambiguous.

Our study suggests a correlation between the presence of tumor-infiltrating lymphocytes and *CTLA4* promoter methylation. These findings are consistent with our recently published methylation data from peripheral blood leukocytes. Among different subtypes of immune cells, CD4^+^ and CD8^+^ T cells, which play a key role in tumor control, were associated with a significantly lower methylation level in the *CTLA4* gene [25]. Consequently, melanoma samples with a lower *CTLA4* promoter methylation level might exhibit a certain immune cell infiltration pattern contributing to response to anti-CTLA-4 immunotherapy. Moreover, our study showed no correlation between *CTLA4* promoter methylation and CTLA-4 protein expression in melanoma. Since we have previously shown a strong inverse correlation between methylation and mRNA expression, this finding needs further investigation, ideally including additional CpG sites since *CTLA4* DNA methylation highly depends on the sequence context [16, 25]. Hypothetical explanations are expression of different CTLA-4 isoforms, posttranslational modifications (e.g., glycosylation), inter- and intratumorally different CTLA-4 turnover rates, and the small sample size. We performed IHC CTLA-4 analysis using a CE IVD-certified monoclonal anti-CTLA-4 antibody intended for in vitro diagnostic. Developed in compliance with In-vitro Diagnostic Directive (IVDD)/Directive 98/79/EC, we expect a high specificity of this antibody which we confirmed using tonsillar tissue as positive control. However, the epitope of this antibody has not been disclosed by the manufacturer, and results regarding the effect of posttranslational modifications on the antibody binding affinity are not publicly available. Four different CTLA-4 protein isoforms can be generated by alternative splicing: full-length CTLA-4 (exons 1- 4), soluble CTLA-4 (without exon 3), ligand-independent CTLA-4 (without exon 2, murine only), and an isoform using only exons 1 and 4 [26]. CTLA-4 is primarily an intracellular antigen and its surface expression is characterized by restricted trafficking to the cell surface and a rapid internalization. Intracellular CTLA-4 is associated with trans-Golgi network and is found in endosomes, secretory granules, and lysosomal vesicles. The regulation of CTLA-4 surface expression might be influenced by glycosylation [26]. The complex spatial distribution, alternative exon usage, and glycosylation might impair an accurate IHC detection. In concordance, no reports have been published that suggest CTLA-4 protein expression as a predictive biomarker for anti-CTLA-4 ICB which is in line with our findings.

The main limitations of our study are the small sample size, the heterogeneity of included patients regarding clinical and molecular features (cutaneous/non-cutaneous, *BRAF*-mutated/wild-type, sample origin [primary tumor/distant metastases/lymph node metastases], pre-treatment), and the analysis of only a limited number of CpG sites that are targeted by the used qMSP assay.

Our study directly links DNA promoter methylation of an immune checkpoint to response to blockade of this particular immune checkpoint. Despite the limited clinical significance of the outdated anti-CTLA-4 ICB monotherapy, our results are of importance for mechanism-driven biomarker strategies in the context of immunotherapies, especially since the protein expression of immune checkpoints only insufficiently predicts response to ICB.

## Availability of data and material

The data that support the findings of this study are available from the corresponding author upon reasonable request.
